# A Case of Shoulder Presentation

**Published:** 1873-05-15

**Authors:** G. D. Mitchell

**Affiliations:** Darwin, Ill.


### A CASE OF SHOULDER PRESENTATION.

BY G. D. MITCHELL, M.D., DARWIN, ILL.

July 23d, 1867, I was called to see M., in consultation with Dr. S. Jumper. From him I got the following history of the case : Some 48 hours previous he had been called to visit the case, but the labor pains were so slight that he returned home. He was sent for the next day, and, some ten hours previous to my arrival, she was delivered of a healthy child. Supposing from appearance, that there was another child, he proceeded to make examination per vaginum, but could not reach the presenting part. Labor pains did not return for something like an hour. When the pains returned, an examination revealed the fact that he had a case of shoulder presentation. Dr. Jumper made an attempt to turn the child, but the labor pains were so powerful that he could not succeed in reaching the feet. When I arrived the pains were still strong, and shoulder well pushed forward down in the pelvis. The shoulder presented with the back of the child towards the pubis, or scapula pubic position of the left shoulder. I made four unsuccessful efforts to secure the feet and bring them down. The tonic contraction was so complete, and the labor pains so violent, and the waters so completely evacuated, that it was impossible to secure the feet.

Opiates and relaxants had no effect upon the uterine contractions. It was impossible in this case to perform cephalic reversion, or bring down the head of the child. Could the child be born in this position? As in twins, the child was small, and favorable to expulsion in this position. Some authors say that sometimes spontaneous evolution, or version, may take place. But the vital powers of the patient were fast failing. The pulse was becoming small and feeble. I proposed to Dr. Jumper to remove the child's head—or, as it is called, decapitation—and bring down the body, and afterwards deliver the head. We agreed to send for Dr. D. O. McCord, for further counsel; but before he arrived we thought further delay was dangerous, and would be jeopardizing the life of the patient, and I proceeded to take off the child's head. This I did by passing a blunt hook around the neck of the child, well wrapped with soft muslin, and taking a long-bladed pocket-knife, wrapped well with soft cloth to within half an inch of the point of the blade, introducing it upon my finger to the neck of the child, and, at the same time, Dr. Jumper drawing upon the hook, to keep the parts within reach. I succeeded in cutting off the child's head, close, of course, to the shoulders, and pushing it back. The labor pains were feeble, and it required some traction on the arm and shoulders of the child to extract it ; but it was delivered in a very few minutes.

At this juncture Dr. McCord arrived, and approved our course. We now proceeded to bring the vertex of the head down, and opened it, letting out the brains, and, introducing the hook into the aperture, succeeded after some time in delivering the head.

There was much difficulty in delivering the after-birth (placenta), from irregular contraction of the womb—similar to hour-glass contraction; and it was some two or three hours before it was accomplished.

The shock to the nervous system was great, and the prostration following lasted several days; but she recovered without any inflammatory symptoms.

I have reported this case for the purpose of making a short report on shoulder presentations in general, and giving my experience in the treatment of these cases; and to urge upon the Society the great importance of being fully prepared and posted to manage shoulder presentations. There are few cases in the practice of medicine in which the reputation of the physician and the safety of the patient are so much at stake as in shoulder presentation. How important it is, then, to have a thorough knowledge of such cases. I will not speak of the different positions, mechanism, diagnosis and prognosis, and the full treatment of shoulder presentations, but give the different modes by which relief is obtained and the mother's life saved.

First. The first is, so authors tell us, the child may be born by duplication, where the child is born with the head doubled upon the body; or, as some have confounded, spontaneous evolution, or version, with duplication, in which the body of the child, if forced down, passing the head, and the nates present at the vulva, and the child delivered as in nates presentation. I have never seen a case of this kind, and believe it hazardous to wait for such a result.

Second. The second is by cephalic version that is to bring down the head of the child in place of the shoulder, converting it into a vertex presentation. Cephalic version is mentioned by authors, but not recommended. It is said to apply to a very small number of cases. I have had five cases of shoulder presentations, in two of which I succeeded in bringing down the head. If the labor is not strong, the shoulder high up, and the time seized for the operation while the waters are escaping, I think it may be performed in many cases. I think it should always be tried, as it does not interfere with pelvic version, if unsuccessful.

Third. The third, pelvic version, or bringing down the feet. This is the most common mode of procedure, and should be performed immediately if cephalic version has failed. This cannot always be performed. We may be called too late, or the contractions are so strong and the waters so completely evacuated from the first that it is impossible to reach the feet.

I have performed pelvic version in two cases, and failed in the one reported above. If the feet cannot be brought down, all that is left us is the

Fourth method of procedure, the lacerating of the child's trunk, to enable us to extract it doubled upon itself, in imitation of the natural process of duplication; or,

Fifth, by decapitating it, in order that the body and head may be separately extracted, as was performed in the case reported above.

I think decapitating the child is far preferable to eviscerating it. When the contents of the chest and abdomen are removed, the body of the child is still so large that the difficulty is by no means all removed. The head and body still must pass out doubled together. To remove the head, the body and head will pass out separately.

The objection to decapitating the child is the supposed difficulty of removing the head afterwards. It appears to me that it is a much easier operation to remove the child's head than to open the chest and remove the lungs and intestines. In the case reported it was no difficult job to open the child's head, and, when opened, the contents escaped immediately. Neither was there any difficulty in extracting the head, by aid of the hook.

The question I wish to ask the Society is, Was this a proper course of procedure, after failing to turn the child by bringing down the feet? Was it good practice to remove the child's head? Immediate help, immediate interference with the natural progress of labor was demanded, beyond all doubt.

